# Auto/paracrine factors and early Wnt inhibition promote cardiomyocyte differentiation from human induced pluripotent stem cells at initial low cell density

**DOI:** 10.1038/s41598-021-00763-z

**Published:** 2021-11-02

**Authors:** Minh Nguyen Tuyet Le, Mika Takahi, Kiyoshi Ohnuma

**Affiliations:** 1 Department of Bioengineering, Nagaoka University of Technology, 1603-1 Kamitomioka, Nagaoka, Niigata, 940-2188 Japan; 2 Department of Science of Technology Innovation, Nagaoka University of Technology, 1603-1 Kamitomioka, Nagaoka, Niigata, 940-2188 Japan

**Keywords:** Cell biology, Developmental biology, Stem cells, Cardiology

## Abstract

Cardiomyocytes derived from human induced pluripotent stem cells (hiPSCs) have received increasing attention for their clinical use. Many protocols induce cardiomyocytes at an initial high cell density (confluence) to utilize cell density effects as hidden factors for cardiomyocyte differentiation. Previously, we established a protocol to induce hiPSC differentiation into cardiomyocytes using a defined culture medium and an initial low cell density (1% confluence) to minimize the hidden factors. Here, we investigated the key factors promoting cardiomyocyte differentiation at an initial low cell density to clarify the effects of cell density. Co-culture of hiPSCs at an initial low cell density with those at an initial high cell density showed that signals secreted from cells (auto/paracrine factors) and not cell–cell contact signals, played an important role in cardiomyocyte differentiation. Moreover, although cultures with initial low cell density showed higher expression of anti-cardiac mesoderm genes, earlier treatment with a Wnt production inhibitor efficiently suppressed the anti-cardiac mesoderm gene expression and promoted cardiomyocyte differentiation by up to 80% at an initial low cell density. These results suggest that the main effect of cell density on cardiomyocyte differentiation is inhibition of Wnt signaling at the early stage of induction, through auto/paracrine factors.

## Introduction

Heart diseases including coronary artery disease and heart attack, result in cardiomyocyte death and damage to heart function^1^. One of the difficulties in treating heart diseases is that cardiomyocytes do not proliferate and regenerate; thus, cells impaired by heart attacks cannot heal naturally. It is thus necessary to produce cardiomyocytes to compensate for the impaired cells, for example, through transplantation. In recent years, cardiomyocyte production via differentiation from human induced pluripotent stem cells (hiPSCs) has attracted increasing attention. Patient-derived hiPSCs possess the same genetic information and do not cause immune rejection^2^. Therefore, a highly successful rate of transplantation and improved quality of life after surgery can be expected.

Cell density is an important factor that determines cell fate; in particular, cardiomyocyte differentiation is greatly affected by cell seeding density^3,4^. Since many studies have reported that an initial high cell density promotes cardiomyocyte differentiation, culturing at an initial high cell density is recommended to direct cell differentiation towards a cardiac fate. For example, cells are plated at 1–4 × 10^5^ cells/cm^2^ and allowed to reach confluence for several days before cardiac induction^5–8^. Indeed, the starting seeding cell density is critical for cardiac differentiation and may require optimization for different cell lines^9^. However, the effect of cell density at each culture stage is unclear. In addition, cardiomyocyte differentiation is induced using Wnt activators followed by Wnt inhibitors^10,11^. Further, it is well known that cardiomyocytes differentiate at the anterior region of the mesoderm, which receives TGF-β and Wnt signaling inhibitors secreted by the anterior visceral endoderm in vivo^12,13^. Thus, the cell density effects might actually be Wnt-modulating factors secreted by the cells themselves (auto/paracrine factors).

In this study, we tested our hypothesis that auto/paracrine factors and Wnt-modulating signals are the primary effects of high cell density during cardiomyocyte differentiation from hiPSCs. To clarify the effects of cell density, we have previously established a method to induce cardiomyocytes from hiPSCs at an initial low cell density (1% confluence)^14^. However, low differentiation efficiency was obtained with only 4% cardiac troponin T (cTnT) expression. We hypothesized that a low cell density would lack certain essential factors required for cardiac differentiation, as compared to high cell densities. Therefore, we examined whether employing a co-culture with high-density cells could improve the differentiation efficiency in low-density cultures. We also compared the expressions of anti-cardiac genes and key cardiac genes during cardiac differentiation using low- and high-density cultures. This study enables an understanding of the key role of seeding density and the importance of time in modulating Wnt signaling for determining the cardiac differentiation efficiency.

## Materials and methods

### hiPSC culture

The KOSM4 hiPSC line, established using a SeVdp-iPS vector^14,15^, was used for all experiments unless otherwise stated. The cells were plated on a fibronectin-coated dish with serum-free medium and were maintained in an ESF9a-based medium with daily medium changes^14^. ESF9a medium consisted of F7 medium (Table S1) with 10 μg/mL insulin (19278-5ML, Sigma-Aldrich), 10 ng/mL basic fibroblast growth factor (rhFGF-2, D2222, Katayama Chemical TTD.), and 2 ng/mL activin A (GFH6-1000, R&D Systems). When the cells reached 80% confluence, they were passaged using TrypLE Select (1 ×) (12563-011, Thermo Fisher Scientific) or TrypLE Express (12604-013, Thermo Fisher Scientific).

### Cardiomyocyte differentiation

Cardiomyocyte differentiation was performed using an established protocol^14^. To maintain pluripotency, hiPSCs were cultured in dishes coated with 5 μg/cm^2^ fibronectin (F1141-5MG, Sigma-Aldrich) using ESF9a medium in the pre-culture stage. Cells were plated at 5 × 10^3^ cells/cm^2^ one day before induction. Cardiomyocyte differentiation was co-induced by a 24-h treatment with 3 µM CHIR99021 (CHIR, 034-23103, R&D Systems) and 10 ng/mL activin A in F7 medium with insulin. Wnt/β-catenin signaling was inhibited at 1–3 days, 2–4 days, or 3–5 days after the induction of differentiation by replacing the medium with RPMI-1640 medium (189-02025, Wako) containing 2% B27 supplement lacking insulin (A18956-01, Thermo Scientific) and 5 or 10 μM IWP2 (3533, Wako, 3533/10, Nacalai tesque) to inhibit Wnt/β-catenin. The culture medium was changed every other day until day 14, and 200 μg/mL insulin was added to the medium from day 7 of the culture.

### Co-culture of high- and low-density cells for cardiomyocyte differentiation

Low-density cells seeded in a 12-well plate were co-cultured with high-density cells plated in a Transwell insert (Thincert cell culture insert, Greiner, 665640). The surface of the Transwell insert is a translucent PET membrane with a pore size of 0.4 μm; this allows for the diffusion of soluble factors from the Transwell insert to the 12-well plate located underneath. The 12-well plates were coated with fibronectin at a concentration of 5 μg/cm^2^. Coatings are not required for Transwell inserts. However, it is necessary to condition the Transwell inserts by covering the entire surface of the membrane with the cell culture medium prior to cell seeding, in order to improve the attachment and spreading of cells. The cells were seeded at a low density of 5 × 10^3^ cells/cm^2^ in the 12-well plate or at a high density of 2 × 10^5^ cells/cm^2^ in the Transwell insert, one day prior to differentiation. The medium volume was 1.5 mL for the 12-well plates and 1 mL for the Transwell inserts. Furthermore, 4 h after plating and once the cells were attached to the surface, co-culturing was commenced. Cells were induced using 3 μM CHIR and cultured under the protocol previously established for low density differentiation^14^. Fourteen days after induction, the cells were harvested for evaluating the cTnT expression via flow cytometry.

### Quantitative reverse transcription polymerase chain reaction (RT-qPCR)

Total RNA was isolated using ISOGEN II (311-07361, Nippon Gene). The medium was removed, 0.5 mL of ISOGEN II was added to the culture plate, and cell lysates were obtained by pipetting. After adding 0.2 mL of RNase-free water and mixing vigorously, the mixture was allowed to stand at 23–28 °C for 15 min. After centrifugation at 12,000×*g* for 15 min, the supernatant was recovered. An equal volume of isopropanol was added and the mixture was allowed to stand at room temperature for 10 min, followed by centrifugation at 12,000×*g* for 10 min to obtain a precipitate. The precipitate was washed twice with 80% ethanol before dissolving in RNase-free water as the total RNA. The isolated total RNA was reverse-transcribed using a PrimeScrip 1st strand cDNA Synthesis Kit (6110A, TaKaRa). The reverse transcription reaction was performed in a thermal cycler at 42 °C for 60 min, 95 °C for 5 min, and 4 °C for 5 min. After this reaction, cDNA was amplified using a TaKaRa PCR Thermal Cycler Dice (TP 650, TaKaRa) using a PCR Amplification Kit (R011, TaKaRa). Finally, the PCR products were separated on a 3% agarose gel and visualized by staining with ethidium bromide. For qPCR, cDNA was synthesized using a PrimeScript RT reagent Kit (RR037A, TaKaRa). qPCR was performed using TaKaRa SYBR Premix Ex Ta II (Tli RNaseH Plus) (RR420A, TaKaRa) and the Thermal Cycler Dice qPCR apparatus integrated with Real Time System Software (TaKaRa – TP800). The primer sets used for amplification are listed in Table S2.

### Immunostaining

Cardiomyocyte differentiation was performed in 96-well plates. Cells were rinsed with 100 μL of phosphate-buffered saline (PBS) containing 0.5 mM CaCl_2_ and 0.5 mM MgCl_2_ (PBS+/+) and fixed for 20 min with 100 μL of 4% paraformaldehyde. The cells were rinsed with 100 μL of PBS+/+, permeabilized with 100 μL of PBS+/+ containing 0.2% Triton X-100 (020-81152, Kishida Chemical), blocked for 60 min with 10 mg/mL bovine serum albumin (BSA, A8806-1G, Sigma-Aldrich), and incubated overnight with primary antibodies (25 μL) in an immunostaining buffer at 4 °C with shaking. The cells were then incubated with secondary antibodies in an immunostaining buffer for 1 h at 23–28 °C with shaking. The primary and secondary antibodies were used after dilution in the immunostaining buffer. Nuclei were stained with 20 µg/mL 4’,6-diamidino-2-phenylindole dihydrochloride (DAPI,045-30361, Wako). The cells were then imaged using a BZ-8100 microscope (Keyence) and analyzed with Image J software (NIH, Bethesda). The antibody information is listed in Table S3.

### Flow cytometry

After 14 days of cardiomyocyte differentiation, the cells were washed with PBS, detached, and singularized using 0.05% w/v trypsin in 0.53 mM ethylenediamine-*N*, *N*, *N*’, *N*’-tetraacetic acid-4Na solution (343-01883, Wako) and 1 mg/mL DNase (LS002139, Funakoshi). The cells were then fixed in 4% paraformaldehyde for 20 min, permeabilized with PBS containing 0.2% Triton X-100, and blocked with 10 mg/mL BSA. These cells were then incubated overnight at 4 °C with primary cTnT mouse IgG1 antibodies (MA5-12960, Thermo Scientific) at 1:75 dilution. The next day, the cells were washed with PBS and incubated for 30 min at 4 °C in the dark with goat anti-mouse Alexa-488 secondary antibodies (Thermo Fisher) at 1:250 dilution. Unbound antibodies were washed with PBS, and the cells were suspended in 500 µL of PBS containing 1 mg/mL BSA. Prior to analysis, the cell suspension was filtered through a 40-µm cell strainer. The cells were analyzed on a FACSCalibur (BD Biosciences, Franklin Lakes). Antibody information is listed in Table S3.

### Statistical analysis

The data are presented as the mean ± SEM of biologically replicated experiments. Differences between two or more groups were evaluated for significance via multiple comparisons, Dunnett’s method, or the t-test with holm’s correction, calculated using a software (ISBN: 978-4-06-156509-8).

## Results

### Co-culture with high-density cells enhanced the efficiency of cardiomyocyte differentiation from low-density cells

**Figure 1:**
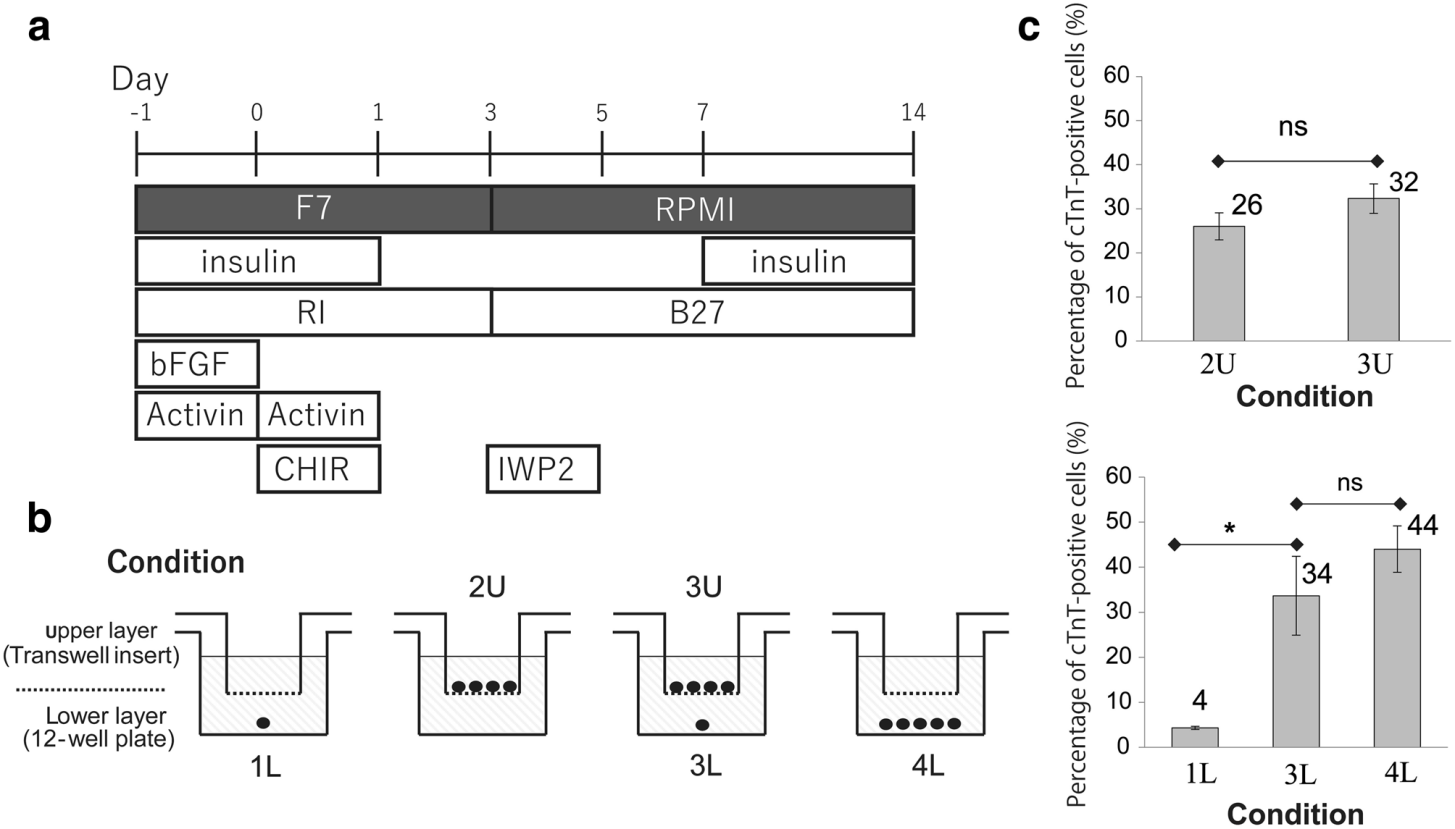
Co-culture of cells plated at an initial high cell density enhanced the cardiomyocyte differentiation efficiency of cells plated at an initial low cell density. (**a**) Schematic of the previously established protocol of cardiomyocyte differentiation^14^. (**b**) Schematic of co-culture experiments. The cells were plated as follows. Cells were plated at a low cell density of 5 × 10^3^ cells/cm^2^ and high cell density of 2 × 10^5^ cells/cm^2^. Condition 1: Only low cell density cells in the lower layer (L indicates lower). Condition 2: Only high-density cells in the upper layer (U indicates upper). Conditions 1 and 2 were used as controls. Condition 3: Co-culture of high-density cells in the upper layer and low cell density cells in the lower layer to assess cell-secreted signals. Condition 4: The same total number of cells at an initial low and high density, as specified in condition 3, was plated on the same surface to assess both cell-secreted signals and cell–cell contact signals. (**c**) The percentage of cTnT-positive cells measured by flow cytometry on day 14. Mean ± standard error (SE), * P < 0.05, t-tests with Holm’s correction, ns: non-significance, n = 3.

A high-density culture may contain factors necessary for cardiac differentiation, whereas low-density cultures may lack these factors. To determine whether the auto/paracrine factors from high-density cells could improve the differentiation performance of a low-density culture, we co-cultured low- and high-density cells through an insert membrane. We used a previously established protocol^14^ (Fig. 1a) to differentiate the cells under various culture conditions. As controls, cells were separately cultured at initial low (5 × 10^3^ cells/cm^2^) and high (2 × 10^5^ cells/cm^2^) cell densities under conditions 1 and 2 (Fig. 1b), respectively. For the co-culture under condition 3, cells were plated at initial high and low cell densities in the upper and lower chambers, respectively. In this condition, only the auto/paracrine factors from high-density cells could diffuse and influence the low cell density cells. To examine the simultaneous influence of auto/paracrine factors and the cell–cell contact signals, condition 4 was designed to contain the same number of cells as the sum of the low- and high-density cells; the cells were cultured on the lower layer. On day 14 after induction, the cardiomyocyte differentiation performance was measured by flow cytometry using cTnT.

By seeding KOSM4 cells at an initial low cell density, 4% cTnT-positive cells were obtained by cardiomyocyte differentiation (Fig. 1c, sample 1L). Co-culture with high-density improved the differentiation efficiency of low-density cells by up to 34% (sample 3L), suggesting the critical role of auto/paracrine factors in cardiomyocyte differentiation. The important role of auto/paracrine factors was confirmed using another cell line (i.e., the 201B7 cell line^2^). Low-density cells co-cultured with high-density cells showed a strong expression of cTnT via immunostaining, whereas the low-density cells alone did not provide any signal (201B7 cell line, Supplementary Fig. S1). Furthermore, the additional effects of cell–cell contact in sample 4L led to 44% cTnT-positive cells, but these results were not significantly different from those of sample 3L. In terms of high-density cells, the separate cultures showed 26% cTnT-positive (2U). The co-culture with low-density cells showed a partly increased differentiation performance to 32% (3U), but this difference was not significant. In summary, these results support our hypothesis that auto/paracrine factors secreted from high-density cells significantly increased the differentiation efficiency of low cell density cultures.

### High cell density inhibits Wnt signaling to inhibit the expression of anti-cardiac genes and promote cardiomyocyte differentiation

To investigate the signals affected by cell density, we compared the expression of key genes relevant to cardiomyocyte differentiation between the initial low and high cell densities. Although *CDX2* and *MSX1* are mesoderm marker genes, these have been reported as anti-cardiac mesoderm genes that suppress cardiac progenitor differentiation^16–18^. Therefore, we investigated the expression of these anti-cardiac mesoderm genes with respect to cardiac progenitor formation at specific cell densities. Cardiac cells were induced at initial low and high densities (5 × 10^3^ and 2 × 10^5^ cells/cm^2^, respectively) using previously established methods^14^. First, Wnt/β-catenin signaling was activated using CHIR from day 0 to day 1 and the early stage mesoderm (anti-cardiac mesoderm) genes, *CDX2* and *MSX1*, were assessed on day 2. Then, Wnt production was inhibited using IWP2 from day 3 to day 5, and the cardiac progenitor cell markers, *ISL1* and *NKX2.5*, were measured.

**Figure 2:**
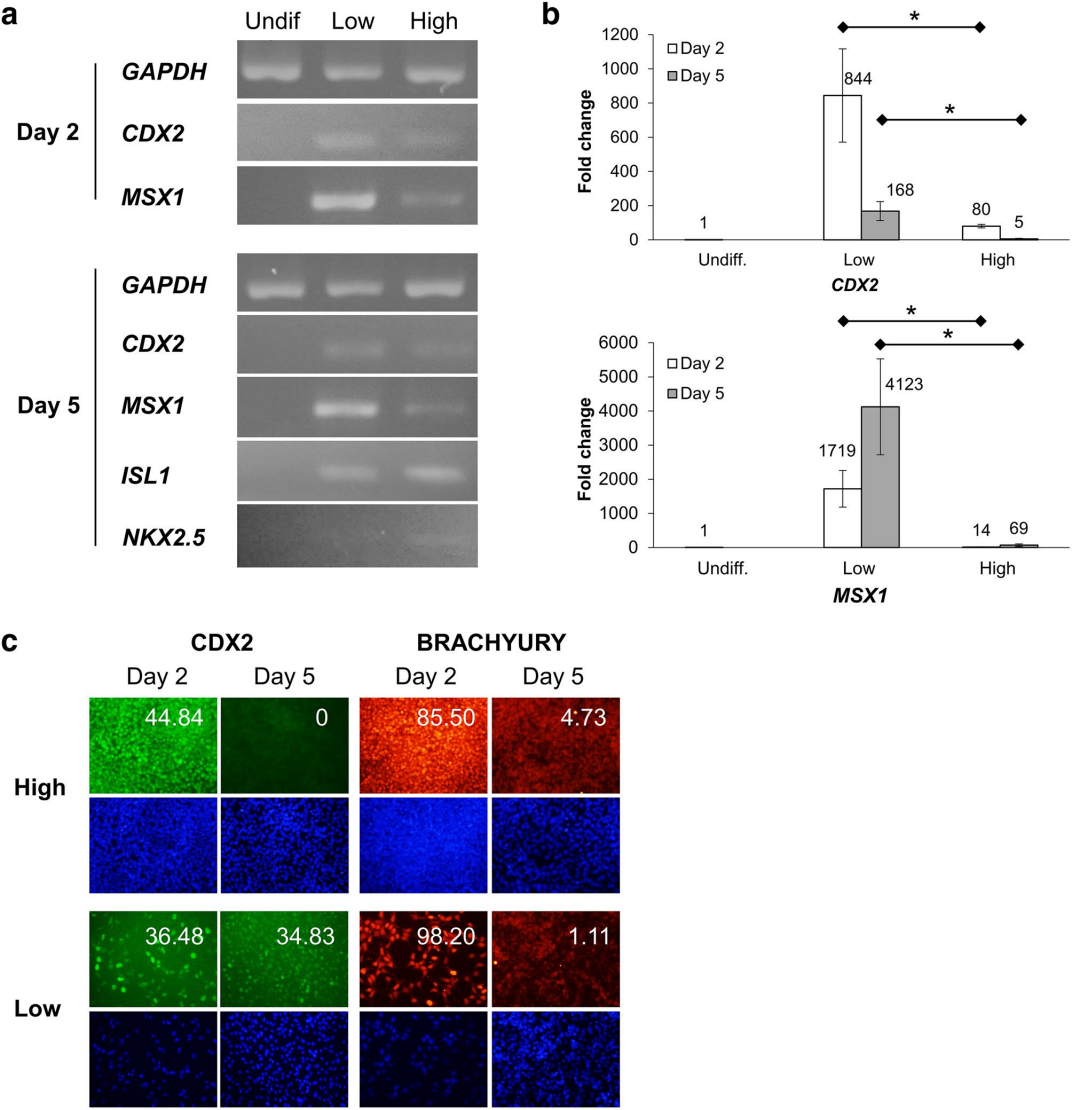
Cells at an initial low cell density expressed a higher level of anti-cardiac mesoderm genes, thereby inhibiting cardiac progenitor expression. (**a**) Expression of anti-cardiac (*CDX2* and *MSX1*) and cardiac progenitor genes (*ISL1* and *NKX2.5*) in undifferentiated cells and in cardiac-differentiated cells at initial low and high densities, on day 2 and 5 by RT-PCR. (**b**) Quantification of anti-cardiac gene expression (*CDX2* and *MSX1*) on day 2 and 5 by RT-qPCR, showing the fold change in comparison with their expression in undifferentiated cells. Mean ± SE, * P < 0.05, t-tests with Holm’s correction, n = 2. (**c**) Immunostaining of cardiac differentiated cells at an initial low and high cell density using CDX2 (green) and BRACHYURY (red) on day 2 and day 5. DAPI (blue) was used for positive control. At an initial low cell density, CDX2 expression was not downregulated but was maintained at high levels on day 5. The numbers indicate the expression of the corresponding markers. Scale bar = 200 μm.

The Wnt/β-catenin signaling markers *AXIN2* and *DKK1* were upregulated after CHIR treatment and downregulated after IWP2 treatment. RT-PCR analysis showed that cells at the initial low cell density expressed higher levels of *CDX2* and *MSX1* compared to those at the initial high cell density (Fig. 2a) at both the early (day 2) and intermediate stages (day 5). The same results were also obtained in 201B7 cell line (Supplementary Fig. S2). On day 5, increased expression of cardiac progenitor genes *ISL1* and *NKX2.5* was observed at the initial high cell density compared to that at the initial low cell density. The expression of the anti-cardiac mesoderm genes was then quantified by RT-qPCR. The initial low cell density cultures showed a higher expression of *CDX2* and *MSX1* compared to the initial high cell density cultures on days 2 and 5 (Fig. 2b). Treatment with IWP2, a Wnt production inhibitor, from day 3 to day 5, lowered the expression of *CDX2* to about 1/4 in cells from the initial low-density culture on day 5, but this was still significantly greater than that at the initial high cell density condition (Fig. 2b). Moreover, longer CHIR treatment (until day 3) increased the expression of anti-cardiac mesoderm genes (*CDX2* and *MSX1*) in high-density cells but decreased the expression of cardiac progenitor genes (*ISL1* and *NKX2.5*, Supplementary Fig. S3). These results suggested that an initial high cell density downregulated the anti-cardiac mesoderm genes, resulting in the activation of cardiomyocyte differentiation.

Next, to evaluate the expression of proteins related to cardiomyocyte differentiation, we performed immunostaining for CDX2 and BRACHYURY (Fig. 2c). CDX2 expression was clear in both initial high and low cell density conditions on day 2. However, on day 5, CDX2 expression was decreased in the initial high cell density condition, whereas it was maintained in the initial low cell density condition (Fig. 2c). Meanwhile, the expression of BRACHYURY, a general mesoderm marker, was decreased on day 5 in both conditions. These results showed that cell density did not affect the expression pattern of BRACHYURY, but it did affect the expression pattern of CDX2. This suggests that cell density affects a subpopulation within mesoderm cells. Based on the gene and protein expression, cardiac induction at the initial low cell density could not suppress anti-cardiac mesoderm expression by the Wnt inhibitor and therefore, failed to induce a high percentage of cardiomyocytes.

We also performed ELISA to clarify whether the Wnt inhibitor protein was supplied as auto/paracrine factors. We measured Wnt inhibitors, such as DKK1, DKK4 and Cerberus, in the supernatant with high-density cells cultures. ELISA data showed that the high-density cells secreted DKK1, DKK4 and Cerberus during the early stages of differentiation; this secretion commenced on day 1 and peaked at day 3 to 5 after induction (Supplementary Fig. S4). These results suggest that a high cell density exhibits a sufficient Wnt signal inhibition effect, which might be induced through auto/paracrine factors such as the secretion of DKK1, DKK4 and Cerberus to suppress anti-cardiac mesoderm genes and facilitate cardiomyocyte differentiation.

### Early addition of Wnt inhibitor improved the efficiency of cardiomyocyte differentiation at an initial low cell density

At an initial low cell density, 5 μM IWP2 application from day 3 to day 5 did not suppress the anti-cardiac mesoderm genes (Fig. 2, day 5), and the efficiency of cardiomyocyte differentiation was very low (Fig. 1, sample 1L). Therefore, we changed the concentration and application timing of IWP2 to increase the efficiency of cardiomyocyte differentiation at an initial low cell density.

**Figure 3:**
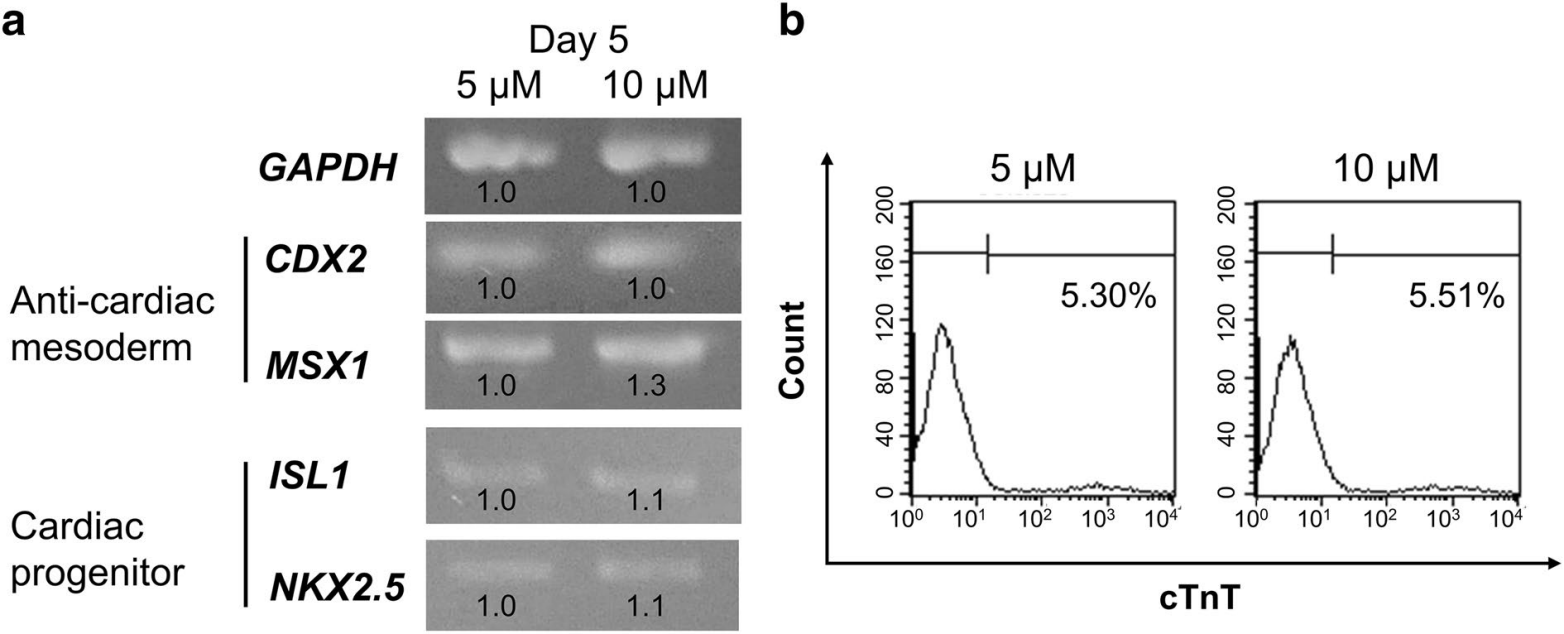
Higher concentrations of Wnt inhibitor did not increase cardiac gene expression at an initial low cell density. (**a**) Expression of anti-cardiac and cardiac progenitor genes on day 5, treated with 5 and 10 μM IWP2 during days 3–5. (**b**) Flow cytometry for cTnT on day 14, in cells treated with 5 and 10 μM IWP2 during day 3–5.

First, we applied a doubled concentration (10 μM) of IWP2 from day 3 to day 5. However, the results showed that doubled concentrations of IWP2 did not change the expression of anti-cardiac mesoderm genes *CDX2* and *MSX1*, of the cardiac progenitor genes *ISL1* and *NKX2.5*, and of the cardiac protein cTnT (Fig. 3a,b). When using 201B7 cell line, doubled concentration of IWP2 also did not change the expression of *CDX2*, *MSX1*, *ISL1*, and *NKX2.5*, or of the Wnt/β-catenin signal genes *AXIN2* and *DKK1* (Supplementary Fig. S5), suggesting that doubled concentration of IWP2 is not effective in increasing cardiomyocyte differentiation.

**Figure 4:**
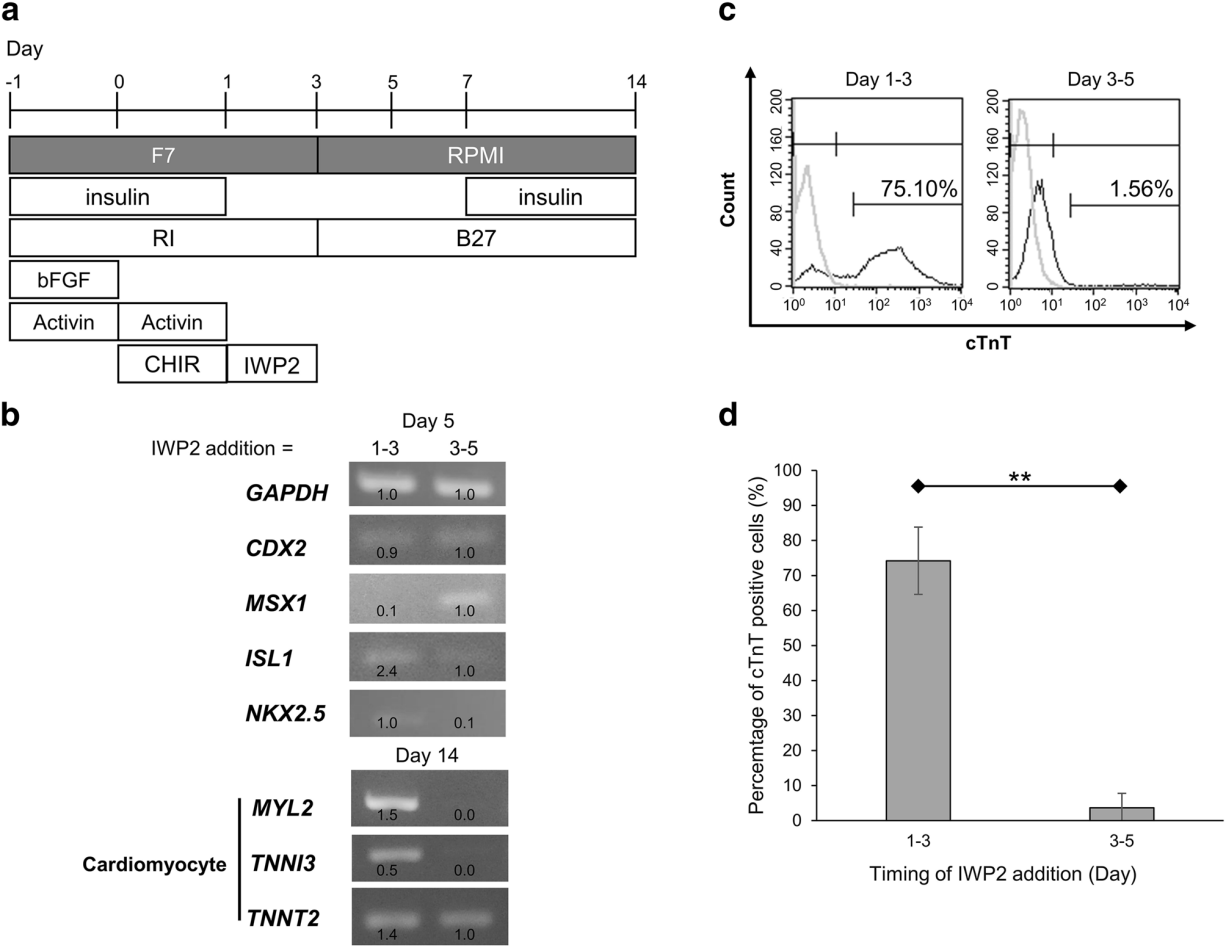
Early addition of Wnt inhibitor during days 1–3 was sufficient to improve cardiomyocyte differentiation efficiency at a low initial cell density. (**a**) Schematic of the new protocol of cardiomyocyte differentiation at an initial low cell density, with early addition of IWP2 from day 1–3. (**b**) Expression of anti-cardiac mesoderm genes *CDX2* and *MSX1* and cardiac progenitor genes *ISL1* and *NKX2.5* on day 5. The expression levels of cardiomyocyte genes (*MYL2*, *TNNI3*, and *TNNT2*) on day 14. IWP2 was added during days 1–3 or days 3–5. (**c**) Flow cytometry of cTnT-positive cells induced by IWP2 treatment during days 1–3 and days 3–5. (**d**) The percentage of cTnT-positive cells under the same conditions as in (**c**). Mean ± SE, ** P < 0.01, t-tests with Dunnett’s correction, n = 4.

Next, we applied IWP2 at an earlier time point (from day 1 to day 3, Fig. 4a). At an initial high cell density, earlier Wnt inhibition (from day 1 or day 2) has been reported as suitable for cardiomyocyte differentiation through the suppression of Wnt signaling^17^. RT-qPCR showed that earlier treatment with IWP2 effectively reduced the expression of anti-cardiac mesoderm genes, thereby increasing the expression of cardiac progenitors and cardiac genes (*MYL2*, *TNNI3*, and *TNNT2*) (Fig. 4b). Moreover, flow cytometry revealed that the addition of IWP2 on days 1–3 induced approximately 80% cTnT-positive cells (Fig. 4c,d). Furthermore, different concentrations of IWP2 also induced cardiomyocytes by early timing of addition (Supplementary Fig. S6). Early addition of IWP2 from day 2 to day 4 also inhibited the expression of anti-cardiac mesoderm genes, promoting the expression of cardiac progenitor genes and cardiac genes (Supplementary Fig. S7), suggesting that earlier application of IWP2 is effective in increasing cardiomyocyte differentiation at an initial low cell density. When using the 201B7 cell line, the early addition of IWP2 from day 1 to day 3, even at a lower concentration of 1–2 μM, induced a robust cTnT expression after 7 days of differentiation in a 96-well plate (Supplementary Fig. S8b). The quantification of cTnT-positive cells using Image J (Supplementary Fig. S8c) yielded the same expression pattern as that for KOSM4 cells cultured in a 12-well plate (Supplementary Fig. S6c). We also applied IWR1, a tankyrase inhibitor that inhibits the Wnt/β-catenin signaling pathway, except for IWP2, a Wnt production inhibitor in 201B7 cell line. IWR1 also induced cardiac cells in low cell density culture, suggesting that this method is versatile and that inhibition of Wnt/β-catenin is important for inducing cardiomyocyte differentiation (Supplementary Fig. S8d).

Collectively, these results showed that upregulation of mesoderm genes followed by prompt downregulation of anti-cardiac mesoderm genes through Wnt signaling inhibition improved cardiomyocyte differentiation at an initial low cell density.

## Discussion

In the present study, we demonstrated that cell-secreted signals, such as auto/paracrine factors, play a more important role in cardiomyocyte differentiation compared to signals arising from cell–cell contact. Moreover, early application of the Wnt production inhibitor IWP2, following Wnt activation, increased the differentiation efficiency (from approximately 4% to 80%) at an initial low cell density (1% confluence). The efficiency of 80%, as determined by the detection of cTnT-positive cells, was comparable to that of other reported methods using an initial high cell density^19–21^.

We used an initial low cell density culture to reveal the effects of cell density in the early stage of cardiomyocyte differentiation. Many protocols induce cardiac cells at a high initial cell density (confluence) by utilizing cell density effects, which are hidden factors for cardiomyocyte differentiation. In contrast, since our protocol used an initial low cell density (1% confluence), the cell density effects were relatively small. Although the cell density effects were reduced in the early stage, they could not be avoided in the later stage of differentiation when the cells proliferated and became confluent. It thus remains a challenge for future research to reduce cell density by re-plating cells at an initial low cell density during the intermediate stage of differentiation and to eliminate the effects of cell–cell interaction in the later stages.

**Figure 5:**
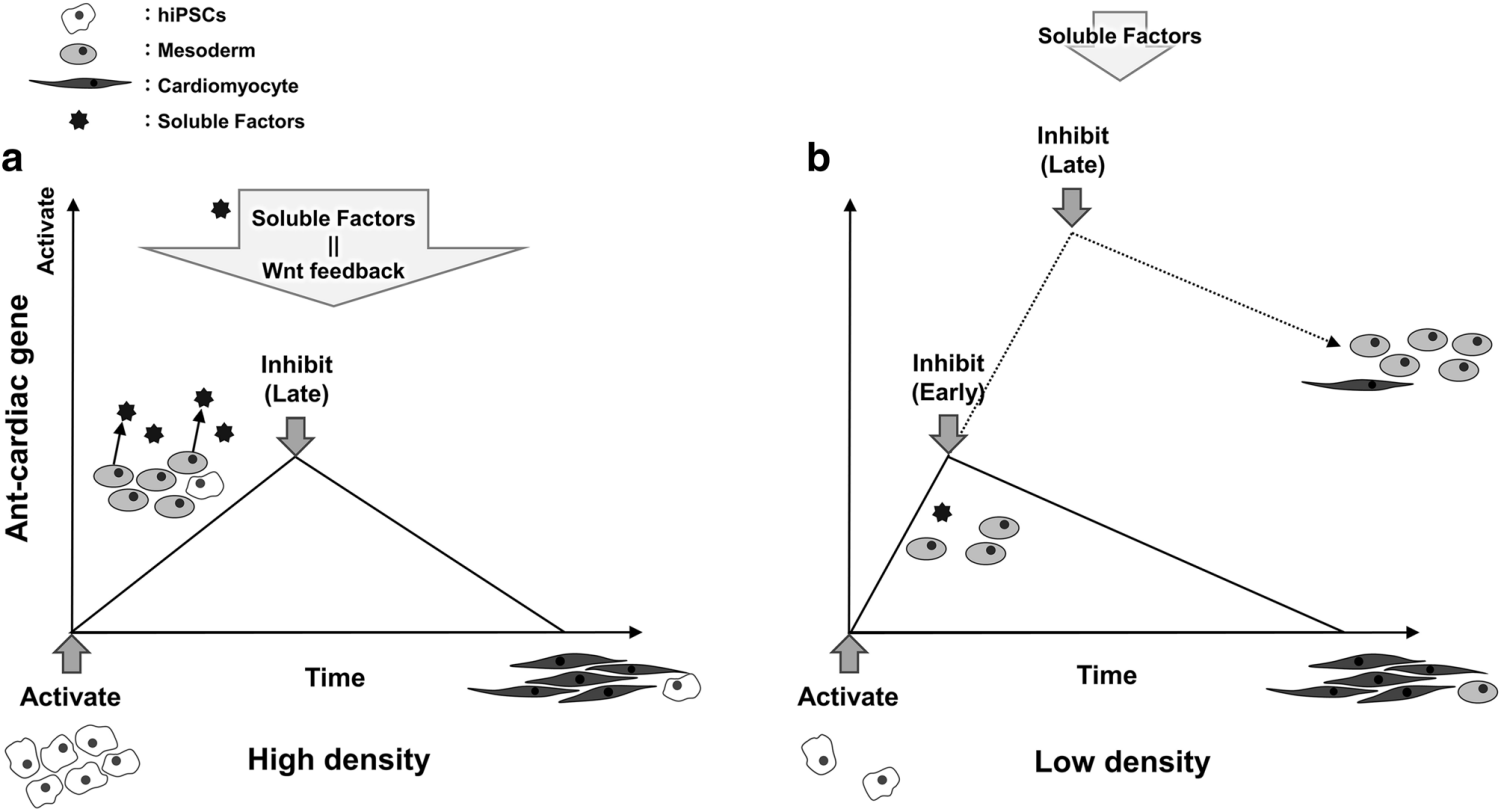
Schematic representation of the results. Cardiomyocyte differentiation at an initial high cell density (**a**) and at an initial low cell density (**b**). Mesodermal cells (gray round) induced by Wnt activators secrete auto/paracrine factors that may suppress Wnt signaling. At an initial low cell density, the suppression provided by the auto/paracrine factor is weak. Early addition of Wnt signaling inhibitors suppresses including non-cardiac mesoderm genes to induce cardiomyocytes.

The cell density effects mainly consist of auto/paracrine factors and cell–cell contact signals. Our co-culture experiment revealed the predominant role of auto/paracrine factors compared to cell–cell contact signals in cardiomyocyte differentiation. Moreover, Wnt inhibitor experiments showed that early application of the Wnt production inhibitor IWP2 or the Wnt/β-catenin signaling inhibitor IWR1 also facilitated cardiomyocyte differentiation. These results suggest that mesoderm cells induced by Wnt activator secrete Wnt signaling inhibitors. It is known that Wnt signaling is regulated via multiple negative feedback mechanisms including auto/paracrine factors^22–24^. These auto/paracrine factors might have the ability of Wnt signal negative feedback to facilitate cardiomyocyte differentiation. At an initial low cell density, early application of Wnt signal inhibitors acts as a substitute for the negative feedback signal supplied by auto/paracrine factors (Fig. 5).

The molecular entity of these auto/paracrine factors is unclear. Candidates include Dickkopf-1 (DKK1), Dickkopf-4 (DKK4) and Cerberus, which are secreted proteins that function as inhibitors of Wnt signaling. DKK1 is also known as a direct target of Wnt signaling and is secreted from PSCs stimulated by CHIR^4,25^. In cardiac development in vivo, Wnt activation is required for mesoderm differentiation, and subsequent Wnt suppression by inhibitors such as DKK1 and Cerberus, secreted from the anterior visceral endoderm, is necessary for cardiomyogenesis^26,27^. Our ELISA results showed that DKK1, DKK4 and Cerberus are secreted by cells and that the concentration peaked at day 3. This is consistent with the results of a previous study, where secretome analysis revealed that the expression of Wnt antagonists (DKK1, DKK4, and CER1) peaked 3 days after the initiation of differentiation^28^. DKK1 is also identified at a higher amount in a conditioned medium of differentiated cells 4 days after the induction of differentiation^29^. Thus, these Wnt inhibitors might act as potential auto/paracrine molecules facilitating cardiac mesoderm induction. This needs to be further clarified in future studies.

In conclusion, we investigated the key factors promoting cardiomyocyte differentiation at an initial low cell density to clarify the causes underlying the cell density effects. Co-culture experiments revealed that auto/paracrine factors and not cell–cell contact signals, played an important role in cardiomyocyte differentiation. Earlier treatment with a Wnt inhibitor efficiently suppressed the expression of anti-cardiac mesoderm genes and promoted cardiomyocyte differentiation by up to 80% at an initial low cell density. These results suggest that the cell density effects of cardiomyocyte differentiation might involve the negative feedback of Wnt signaling by auto/paracrine factors. Future works should investigate whether the early inhibition of Wnt signaling improves the differentiation efficiency in our co-culture system, as well as in disease or “difficult to differentiate” hiPSC lines. Although we tested our system on two cell lines, it is necessary to investigate using other cell lines to demonstrate the reproducibility of the effect. Our protocol may prove useful for analyzing the differentiation of cardiomyocytes in order to understand the developmental process in greater detail without any hidden factors.

## Supplementary Information

Supplementary Information.
