# Supplementary material for: Auto/paracrine factors and early Wnt inhibition promote cardiomyocyte differentiation from human induced pluripotent stem cells at initial low cell density
Source: Sci Rep. 2021 Nov 2;11:21426. doi: 10.1038/s41598-021-00763-z (PMC8563935; doi:10.1038/s41598-021-00763-z)
Supplement: Supplementary file 1 — Supplementary Information. [file 41598_2021_763_MOESM1_ESM.pdf]

Supplementary information

Auto/paracrine factors and early Wnt inhibition promote cardiomyocyte differentiation from human induced pluripotent stem cells at initial low cell density

Minh Nguyen Tuyet Le [1,*], Mika Takahi [2,*], Kiyoshi Ohnuma [1,2,**]

[1] Department of Bioengineering, Nagaoka University of Technology, 1603-1 Kamitomioka, Nagaoka, Niigata 940-2188, Japan

[2] Department of Science of Technology Innovation, Nagaoka University of Technology, 1603-1 Kamitomioka, Nagaoka, Niigata 940-2188, Japan

* These authors contributed equally to this work

** Correspondence should be addressed to Kiyoshi Ohnuma

e-mail: kohnuma@vos.nagaokaut.ac.jp, Tel/Fax: +81-258-47-9454

In supplementary figures S6 and S7, the human induced pluripotent stem cell (hiPSC) line KOSM4 [1, 2] was used. In supplementary figures S1-5 and S8, another human induced pluripotent stem cell (hiPSC) line, 201B7 [3] was used to support the main results.

Cell culture of 201B7

201B7 was obtained from the RIKEN BRC Cell Bank (HPS0063, Tsukuba, Ibaraki, Japan) through the National Bio-Resource Project for the Ministry of Education, Culture, Sports, Science, and Technology, Japan. The cells were plated in serum-free medium (StemFit AK02 N, Ajinomoto, according to the protocol supplied by the manufacturer of the medium) with Y-27632 (Rock inhibitor, final concentration was 5 μM, 036-24023, Wako) and 0.25 μg/cm^2 laminin (iMatrix-511, 387-10131, Wako) [4]. On the following day, the medium was changed to StemFit AK02 N medium without Y-27632 and the medium was then changed daily. When the cells reached 80% confluence, they were passaged using TrypLE Select (1×) or TrypLE express.
The methods and reagent for cardiac cell induction were the same as those described in the main text. In Fig. S8, we used IWR1 (I0161-5MG, Merck), a tankyrase inhibitor to inhibit the Wnt/β-catenin signaling pathway.

PCR and immunostaining

PCR and immunostaining methods were also the same as those mentioned in the main text.

Measurement of protein secretion by ELISA

The cells were seeded at a high density of 2×10^5 cells/cm^2 in the 24-well (DKK1) or 12-well (DKK4, CER1) plate one day prior to inducing cardiomyocyte differentiation. The supernatant was collected and the number of cells were counted immediately before induction (day 0) and at 1, 3 and 5 days after cardiomyocyte differentiation. DKK1 concentration was measured using Human DKK-1 Quantikine ELISA Kit (DKK100B, R&D Systems), Human Cerberus 1 ELISA Kit, (ELH-CER1, Ray biotech) and Human Dkk-4 ELISA Kit (ELH-DKK4, Ray biotech)

according to the protocol supplied by the manufacturers. Briefly, for DKK1, the mixture of assay diluent and sample were incubated for 2 hours at room temperature on a shaker. After each well was rinsed by the wash buffer four times, human Dkk-1 Conjugate was added to each well and the plate was incubated for 2 hours at room temperature on the shaker. After reaction, all wells were rinsed four times and then substrate solution was added to each well and incubated for 30 minutes at room temperature on the benchtop without light. Finally, stop solution was added to each well and determine the optical density of each well using a microplate reader (680, BIO-RAD) set to 450 nm, and the wavelength correction was applied at 540 nm.

For DKK4 and CER1, assay diluent was added to each well. After that, samples were added and incubated for 2.5 hours at room temperature on a shaker. Next, each well was rinsed with the wash buffer four times. Then, biotinylated antibody was added to each well and the plate was incubated for 1 hours at room temperature on the shaker. After the reaction, each well was rinsed four times, streptavidin solution was added to each well and the plate was incubated for 45 minutes at room temperature. After reaction, each well was rinsed four times, TMB one-step substrate reagent was added to each well and the plate was incubated for 30 minutes at room temperature on the benchtop shielded from light. Finally, stop solution was added to each well and the optical density of each well was determined using a microplate reader set to 450 nm.

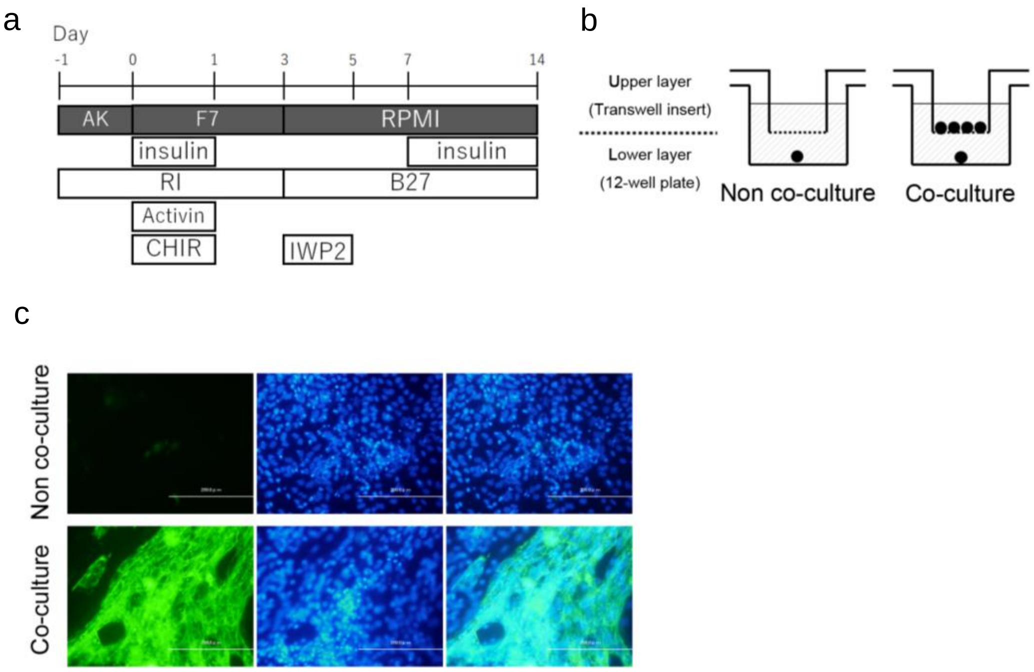

Fig. S1 Co-culture with high-density cells promoted cardiac differentiation in low-density culture for 201B7 cells.

(a) Schematic of cardiac differentiation at an initial low cell density (5×10^3 cells/cm^2).

(b) Schematic of co-culture experiments.

(c) Immunostaining of cardiac-differentiated cells at an initial low using cTnT on day 14. DAPI (blue) was used for positive control. Scale bar = 200 µm.

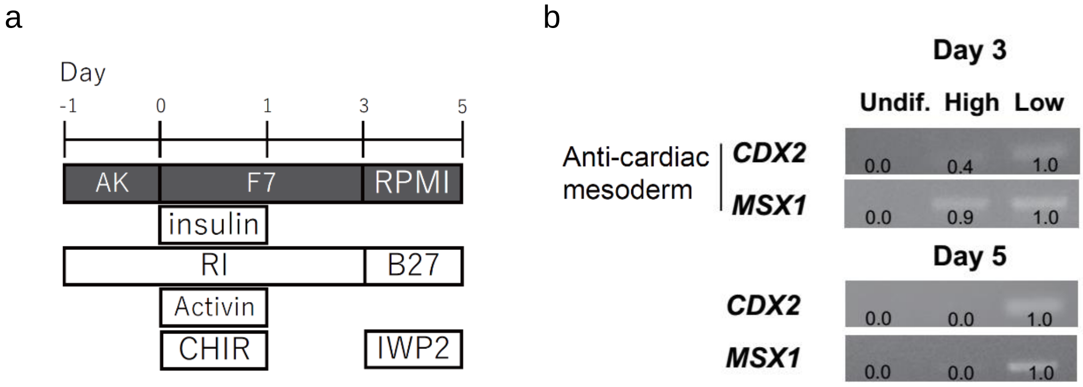

Fig. S2 Expression of anti-cardiac mesoderm genes, *CDX2* and *MSX1*, at an initial low and high cell density in 201B7 cells

(a) Schematic of cardiac differentiation at an initial high (High: 2×10^5 cells/cm^2) and low (Low: 5×10^3 cells/cm^2) cell density, with addition of the Wnt production inhibitor, IWP2 from day 3-5. Differentiation was co-induced by 10 ng/mL activin A and 6 μM CHIR99201.

(b) Expression of anti-cardiac mesoderm genes *CDX2* and *MSX1* on days 3 and 5 by RT-PCR. For semi-quantification, gene expression of low density cell was set as 1.0.

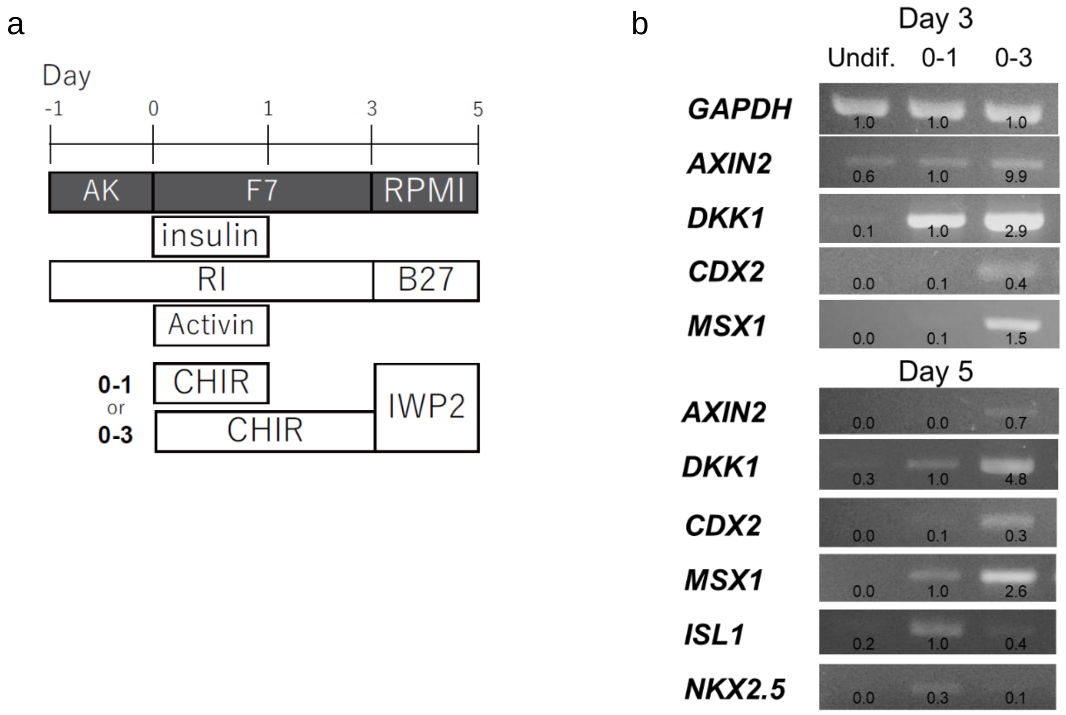

Fig. S3 Gene expression by long-term CHIR addition in 201B7 cells

(a) Schematic of cardiac differentiation at an initial high cell density (2×10^5 cells/cm^2) on day -1. The Wnt activator, CHIR99201 (final concentration: 6 µM), was applied from day to 0-1 or day 0-3 and the Wnt production inhibitor, IWP2 (final concentration: 5 µM), was applied from day 3 to day 5. Differentiation was co-induced by 10 ng/mL activin A.

(b) Expression of Wnt/β-catenin signaling (*AXIN2* and *DKK1*), anti-cardiac (*CDX2* and *MSX1*) and cardiac progenitor genes (*ISL1* and *NKX2.5*) on days 3 and 5 by RT-PCR.

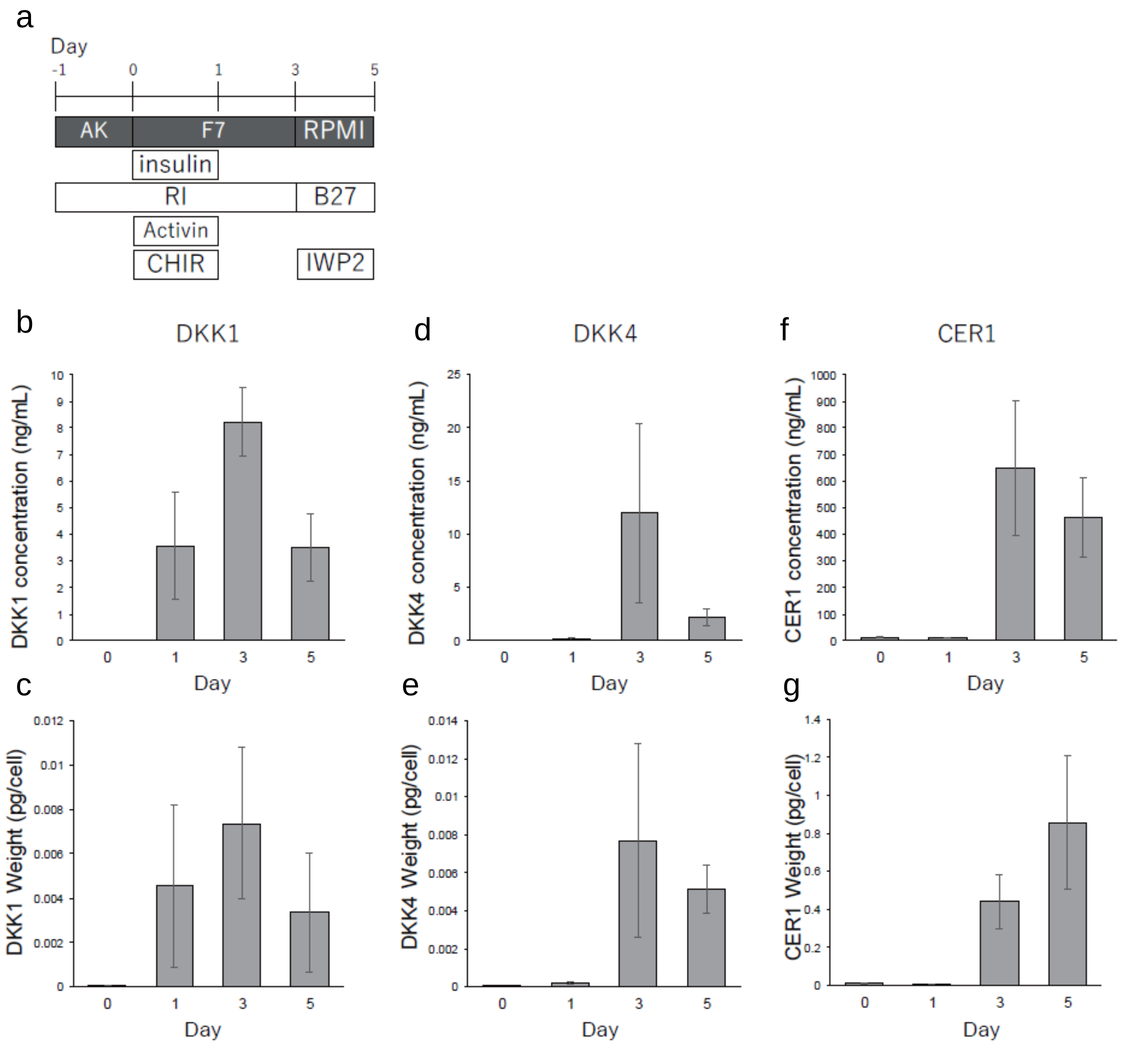

Fig. S4. High-density cells secrete the Wnt inhibitor DKK1, DKK4 and CER1, which peak 3 days after the induction of cardiomyocyte differentiation in 201B7 cells.

(a) Schematic of cardiac differentiation at an initial high cell density (2×10^5 cells/cm^2). Differentiation was co-induced by 10 ng/mL activin A and 6 μM CHIR99201.

(b-g) The graph shows the amount of DKK1, DKK4 and CER1 secreted from one cell or the total concentration of these Wnt inhibitors during cardiac differentiation at a high cell density. Mean ± SE, * $P < 0.05$, Dunnett's multiple comparison, n=4.

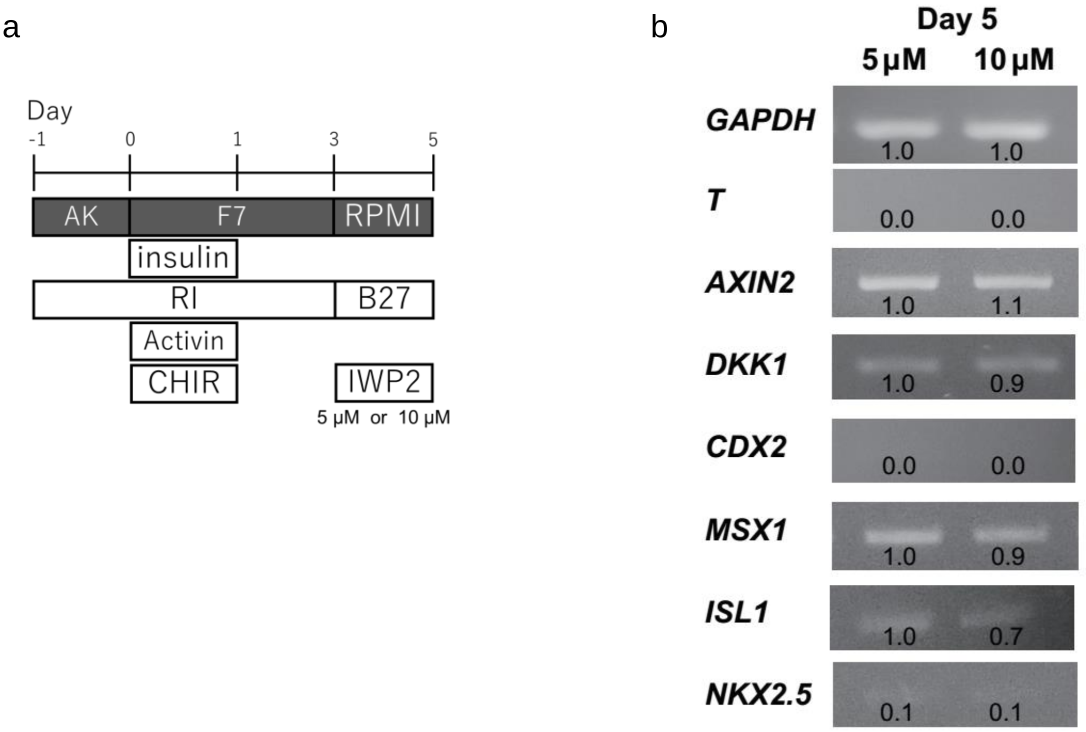

Fig. S5. Doubled concentration of IWP2 did not affect gene expression during cardiac differentiation at an initial low cell density condition in 201B7 cells

(a) Schematic of cardiac differentiation at an initial low cell density (5×10^3 cells/cm^2) on day -1. The Wnt production inhibitor, IWP2 (final concentration: 5 μM or 10 μM), was applied from day 3 to day 5. Differentiation was co-induced by 10 ng/mL activin A and 6 μM CHIR99201.

(b) Expression of mesoderm (*T*), Wnt/β-catenin signaling (*AXIN2* and *DKK1*), anti-cardiac (*CDX2* and *MSX1*), and cardiac progenitor (*ISL1* and *NKX2.5*) on day 5 by RT-PCR.

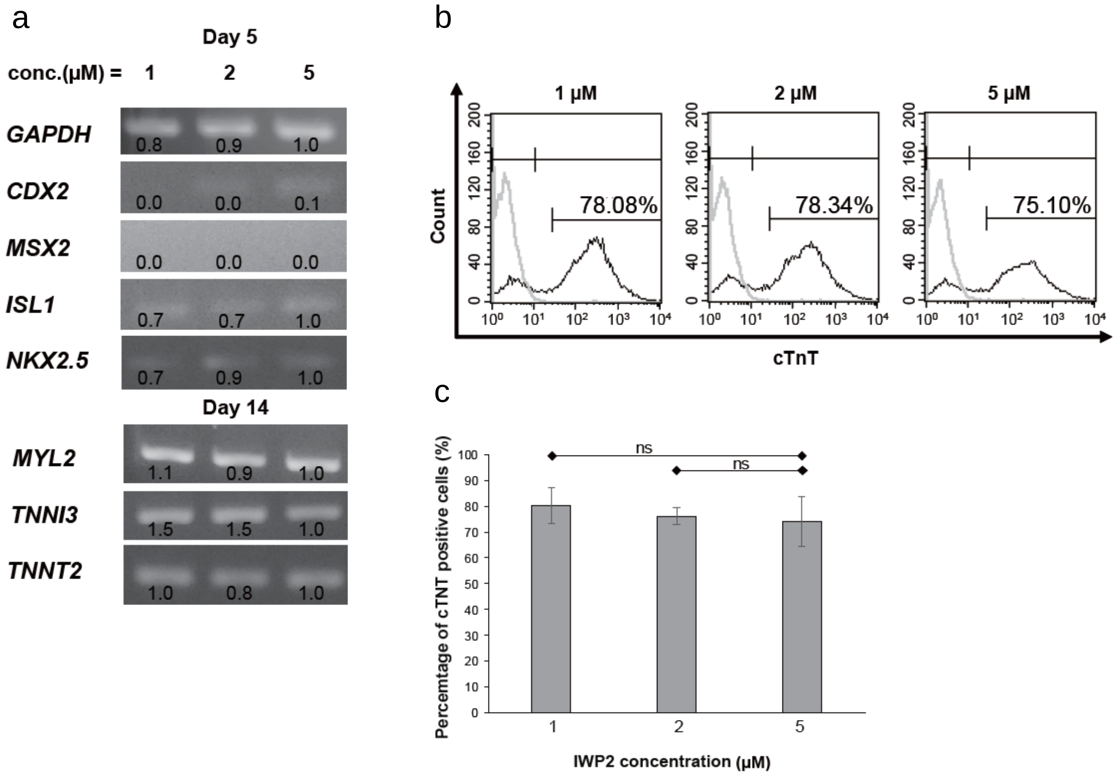

Fig. S6. Even low concentrations of IWP2 induced cardiac differentiation with high efficiency at a low initial cell density in KOSM4 cells

(a) Expression of anti-cardiac mesoderm genes *CDX2* and *MSX1* and cardiac progenitor genes *ISL1* and *NKX2.5* on day 5. The expression levels of cardiomyocyte genes (*MYL2*, *TNNI3,* and *TNNT2*) on day 14. IWP2 was added during day 1–3.

(b) Flow cytometry of cTnT-positive cells induced by IWP2 treatment at each indicated concentration during day 1–3.

(c) The percentage of cTnT-positive cells under the same conditions as (b). Mean ± SE, ** $P < 0.01$, *t*-tests with Dunnett's correction.

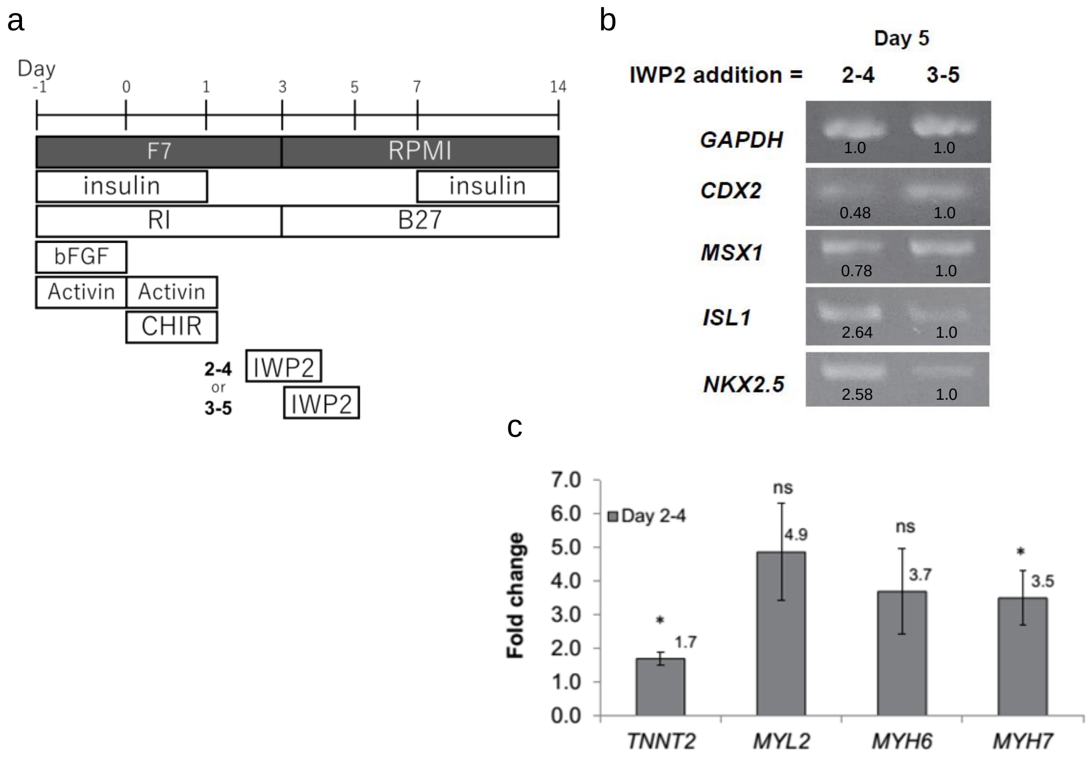

Fig. S7. Early addition of Wnt inhibitor during day 2–4 was sufficient to improve the cardiomyocyte differentiation efficiency at a low initial cell density in KOSM4 cells

(a) Schematic of cardiac differentiation at an initial low cell density (5×10^3 cells/cm^2) on day -1. The Wnt production inhibitor IWP2 (final concentration: 5 μM), was applied from day 3 to 5 or day 2 to 4. Differentiation was co-induced by 10 ng/mL activin A and 3 μM CHIR99201. IWP2 was added on days 3–5 or earlier on days 2–4 at a concentration of 5 μM.

(b) Expression of anti-cardiac mesoderm genes *CDX2* and *MSX1* was examined along with that of cardiac progenitor markers *ISL1* and *NKX2.5* on day 5 by RT-PCR.

(c) Expression of cardiac terminal genes, including cardiac troponin T (*TNNT2*), myosin light chain (*MYL2*), and myosin heavy chain alpha and beta (*MYH*6 and *MYH7*) on day 14 by RT-qPCR. Data represent mean ± SE, n = 3, * $P < 0.05$, *t*-tests with Holm's correction, ns: non-significance.

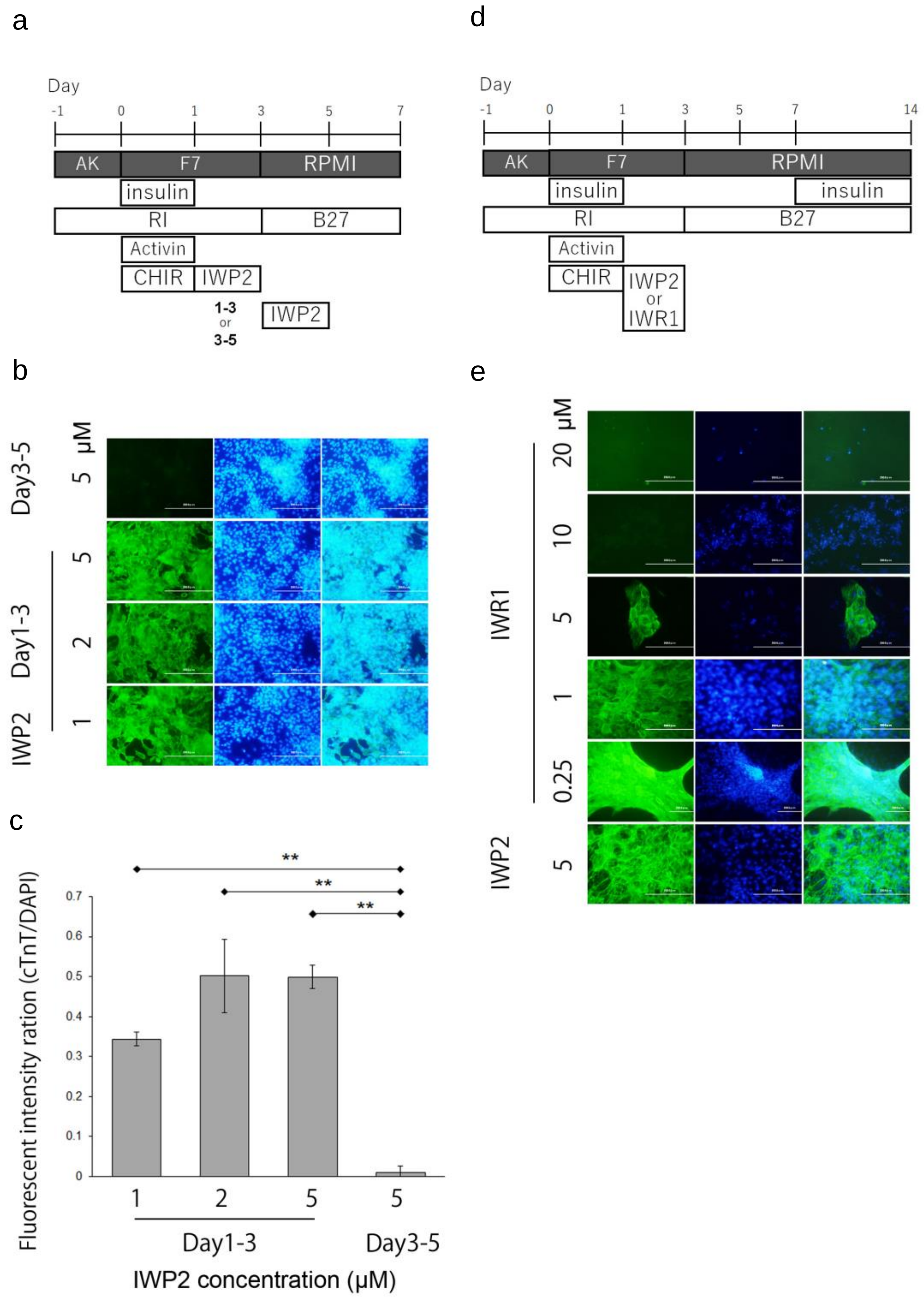

Fig. S8. Early addition of the Wnt/β-catenin signaling inhibitor induces cardiac differentiation at an initial low cell density for 201B7 cells

(a) Schematic of cardiac differentiation at an initial low cell density (5×10^3 cells/cm^2) on day -

1, with the Wnt production inhibitor IWP2 added from days 1–3 (final concentration: 1, 2 and 5 μM) or days 3-5 (final concentration: 5 μM).

(b) Early addition of IWP2 induced cardiac differentiation even at low concentrations at a low initial cell density in 201B7 cells. Cells were fixed on day 7 for immunostaining using cTnT (green) and DAPI (blue). Scale bar = 200 μm.

(c) Quantification of cTnT-positive cells under the same conditions as in (b) using Image J. Mean ± SE, ** $P < 0.01$, t-tests with Dunnett's correction, $n = 3$.

(d) Schematic of cardiac differentiation at an initial low cell density (5×10^3 cells/cm^2) on day -1, with the Wnt production inhibitor, IWP2 (final concentration: 5 μM), or the Wnt/β-catenin signaling inhibitor, IWR1 (Final concentration: 0.25–20 μM) from days 1–3.

(e) The Wnt/β-catenin signaling inhibitor IWR1 was added during the early stages of days 1-3 at different concentrations. Cells were fixed on day 14 for immunostaining using the cardiac differentiation marker cTnT (green) and DAPI (blue). Scale bar = 200 μm.

a

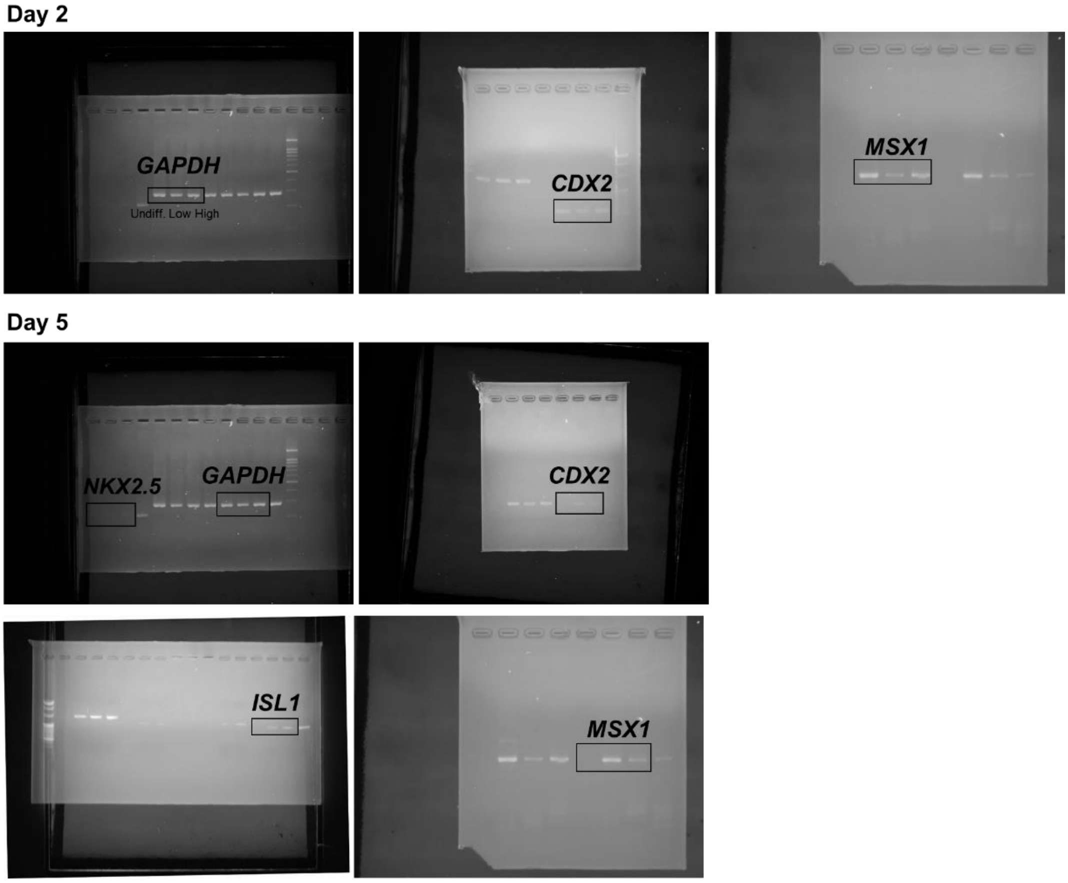
Day 2
GAPDH
Undiff. Low High
CDX2
MSX1
Day 5
NKX2.5
GAPDH
CDX2
ISL1
MSX1

b

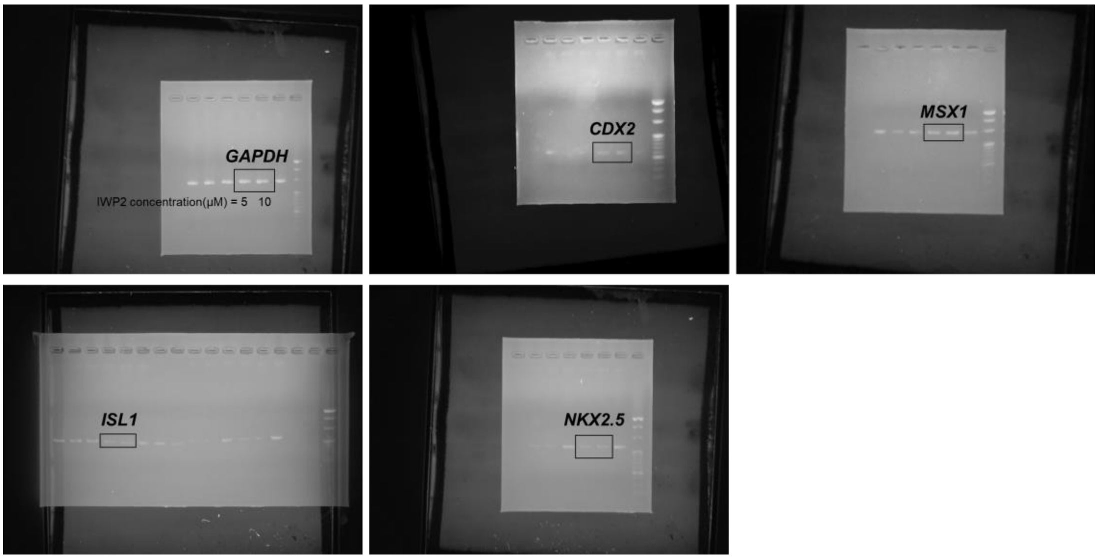
GAPDH
IWP2 concentration(μM) = 5 10
CDX2
MSX1
ISL1
NKX2.5

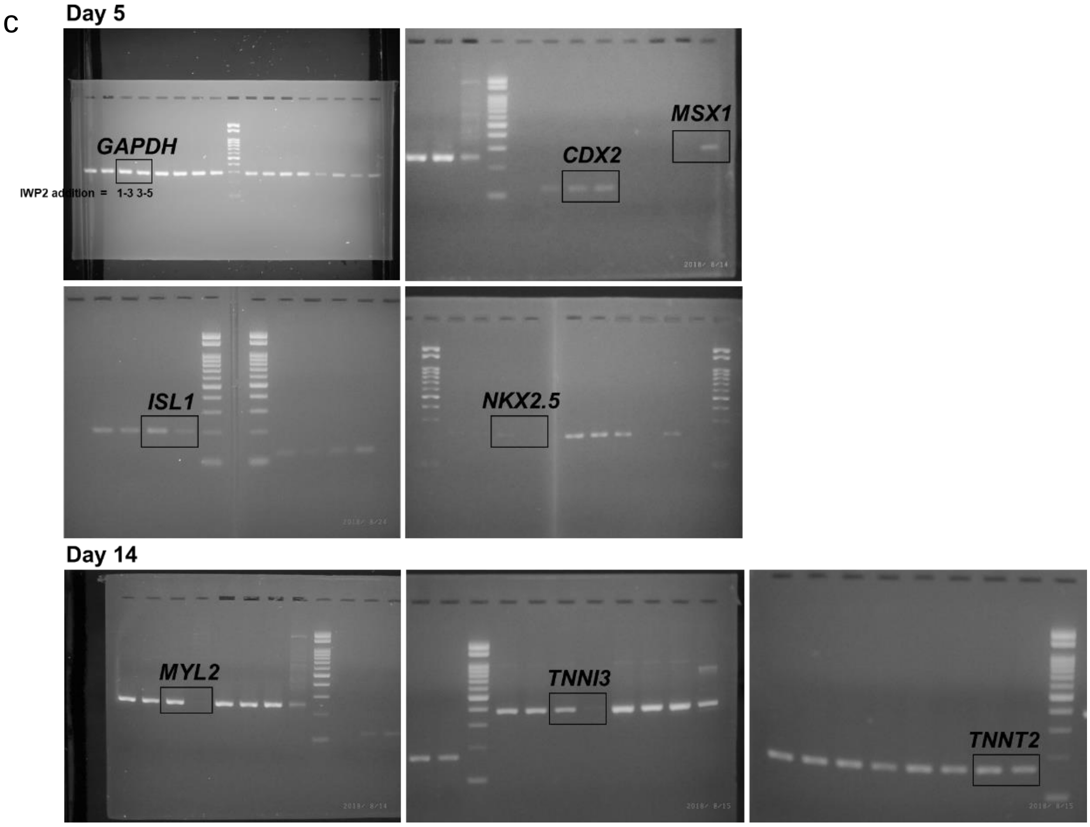
c
Day 5
GAPDH
IWP2 addition = 1-3 3-5
CDX2
MSX1
ISL1
NKX2.5
Day 14
MYL2
TNNI3
TNNT2

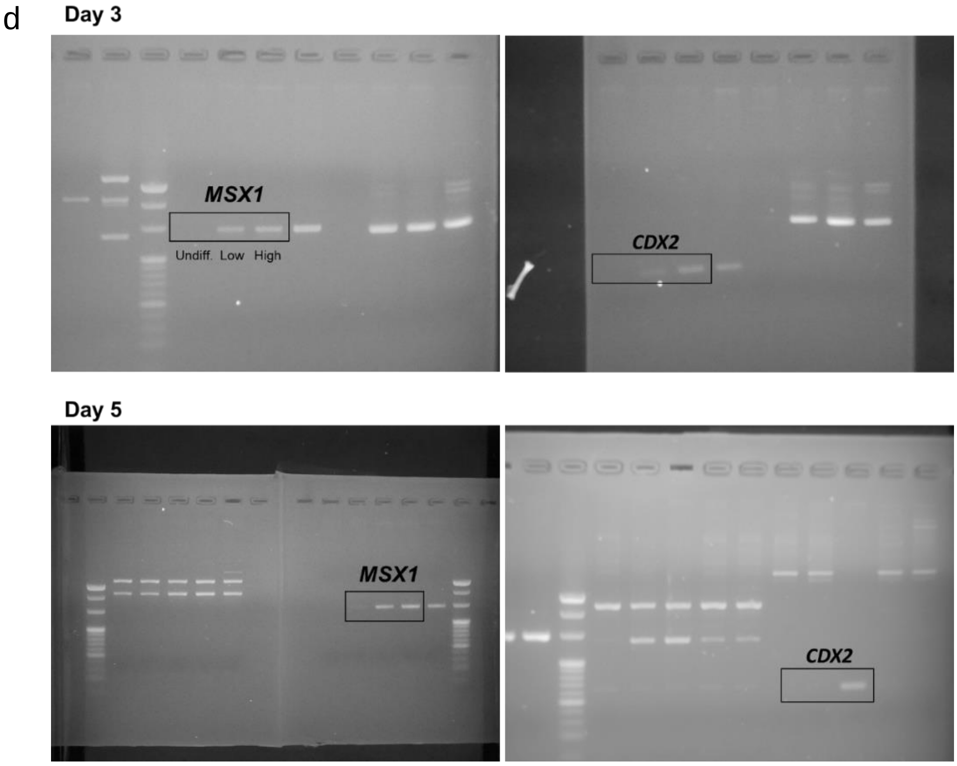
d
Day 3
MSX1
Undiff. Low High
CDX2
Day 5
MSX1
CDX2

e

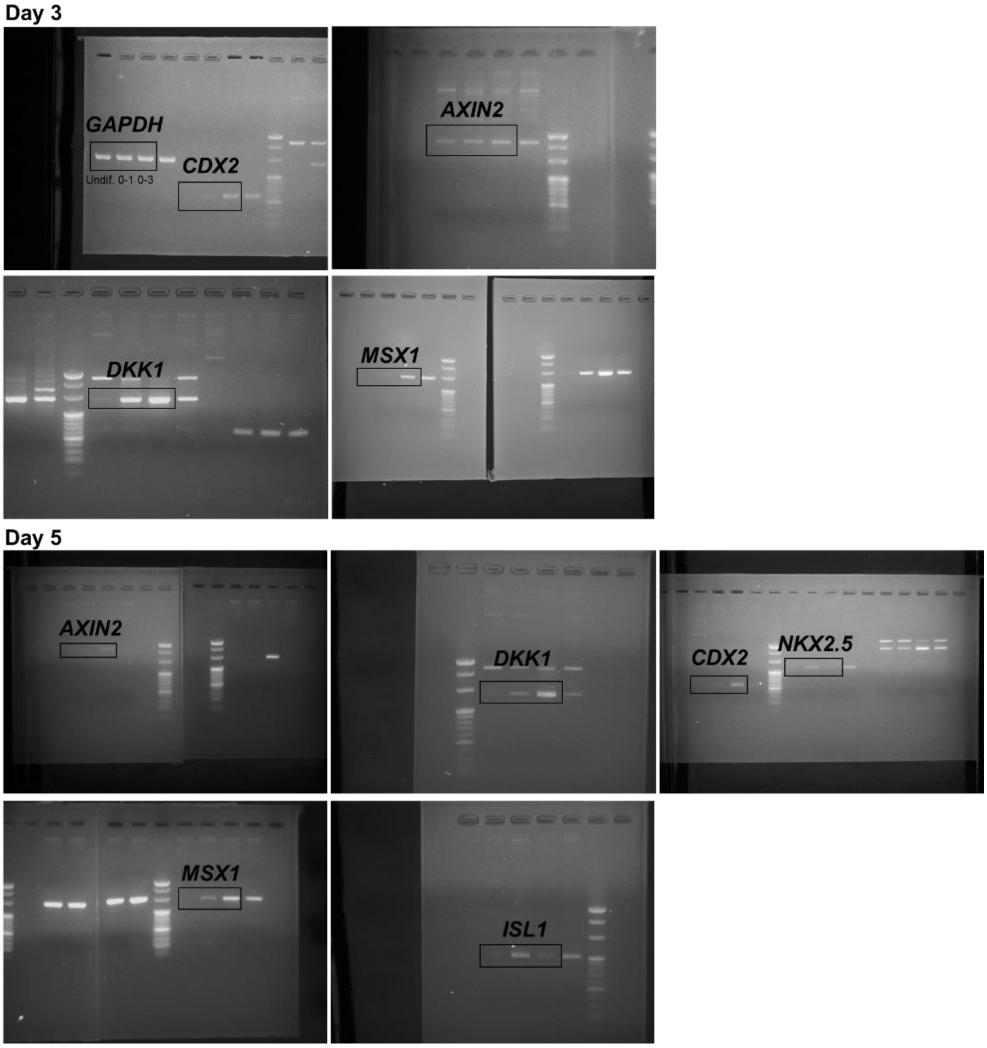
Day 3
GAPDH
Undif. 0-1 0-3
CDX2
AXIN2
DKK1
MSX1
Day 5
AXIN2
DKK1
CDX2
NKX2.5
MSX1
ISL1

f

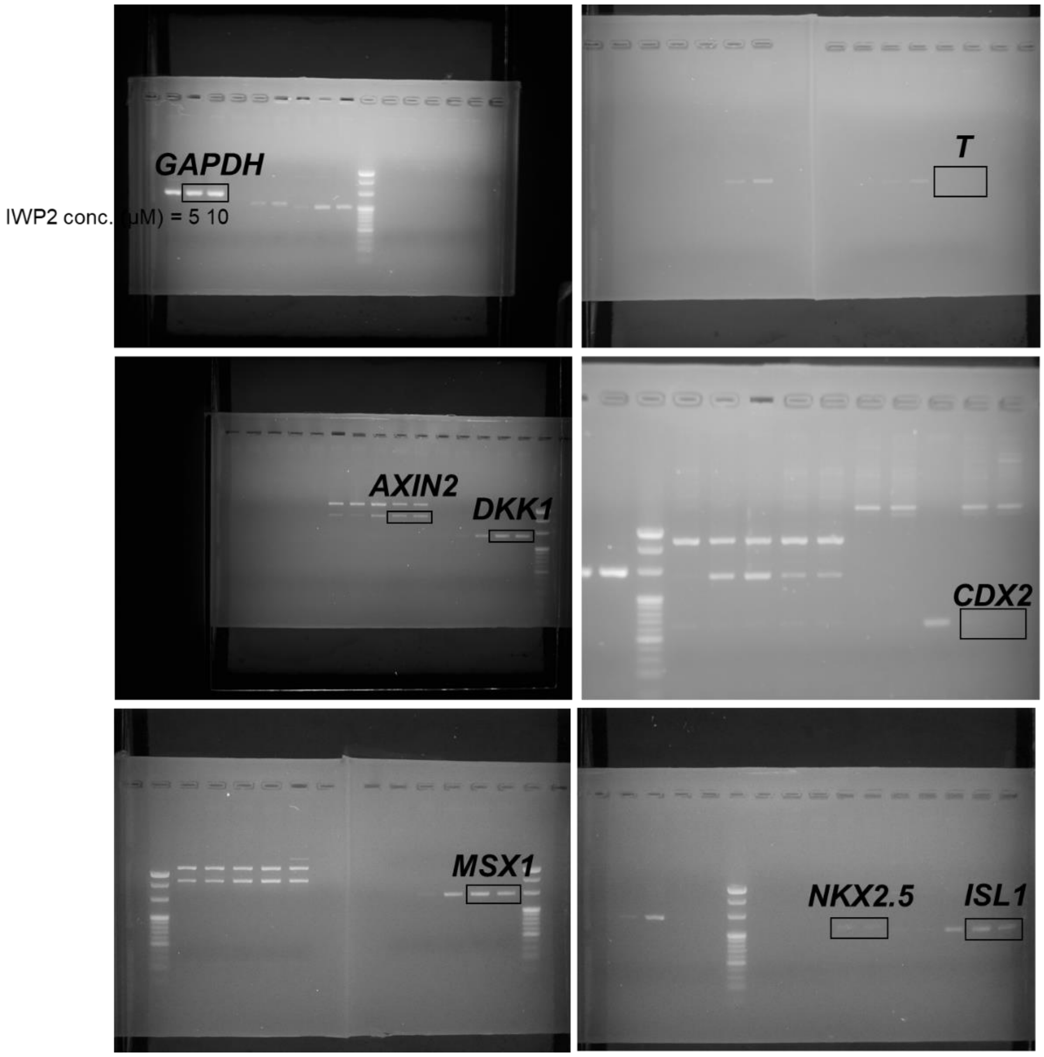

157

158

g

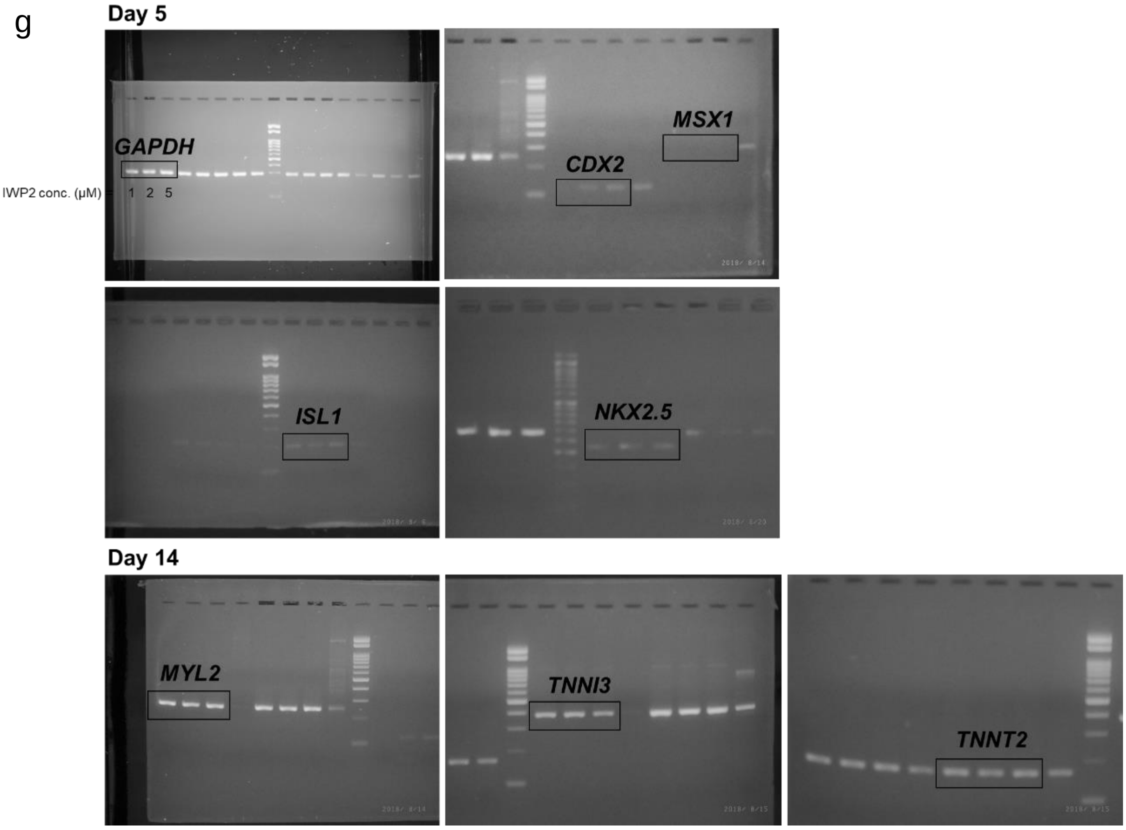

h

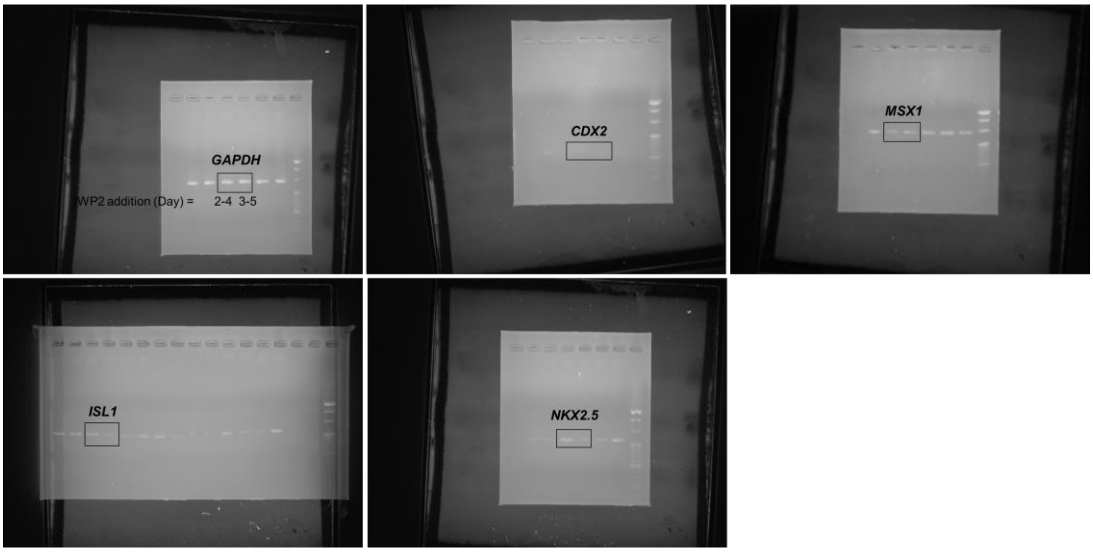

Fig. S9. Full-length gels of electrophoresis

(a) Figure 2.

(b) Figure 3.

(c) Figure 4.

(d) Figure S2.

(e) Figure S3.

(f) Figure S5.

(g) Figure S6.

(h) Figure S7.

Table S1. F7 medium components

Reagent	Final concentration	Manufacturer
mESF basal medium		Wako
Transferrin	5 µg/mL	Sigma-Aldrich
Ethanolamine	10 µM	Sigma-Aldrich
Sodium selenite	20 nM	Sigma-Aldrich
2-mercaptoethanol	10 µM	Sigma-Aldrich
L-ascorbic acid-2-phosphate	100 ng/mL	Wako
Oleic acid (OA)-BSA	OA 9.4 µg/mL, BSA 1 mg/mL	Sigma-Aldrich
Heparin	0.1 µg/mL	Sigma-Aldrich

Table S2. Primer information

Marker		Sequence (5'→3')	Size (bp)	PCR method[a,b,c]	Cycle number
GAPDH Internal control	F	TGACCTGCCGTCTAGAAAAACC	288	3-step – 58°C	30
	R	TGGTCCAGGGGTCTTACTCCTT			
ISL1	F	CACAAGCGTCTCGGGATT	202	3-step – 58°C	30
	R	AGTGGCAAGTCTTCCGACA			
NKX2.5	F	GCGATTATGCAGCGTGCAATGAGT	220	3-step – 70°C	30
	R	AACATAAATACGGGTGGGTGCGTG			
TNNI3	F	CTGCAGATTGCAAAGCAAGA	379	3-step – 58°C	40
	R	CCTCCTTCTTCACCTGCTTG			
TNNT2	F	TTCACCAAAGATCTGCTCCTCGCT	165	3-step – 58°C	40
	R	TTATTACTGGTGTGGAGTGGGTGTGG			
MYL2	F	ACATCATCACCCACGGAGAAGAGA	247	3-step – 58°C	40
	R	ATTGGAACATGGCCTCTGGATGGA			
CDX2	F	GCCAACCTGGACTTCCTGTCA	119	3-step – 58°C	30
	R	TCTGGCTTGGATGTTACACAGACC			
MSX1	F	CCGAGAGGACCCCGTGGATGC	280	2-step	40
	R	GCCTCTTGTAGTCTCTTTGCC			
DKK1	F	GTGCAAATCTGTCTCGCCTG	266	3-step – 70°C	30
	R	GCACAGTCTGATGACCGGAG			
AXIN2	F	GGCTGCGCTTTGATAAGGTC	418	2-step	35
	R	GCCTGGTGTTGGAAGAGACA			

Specific temperatures of 2-step and 3-step conditions:

PCR condition

[a] 2-step cycle: 94°C for 30 s, 68°C for 30 s, 72°C for 5 min, 4 °C

[b] 3-step cycle (58°C): 94°C for 30 s, 58°C for 30 s, 72°C for 40 s, 72°C for 5 min, 4°C

[c] 3-step cycle (70°C): 94°C for 30 s, 70°C for 30 s, 72°C for 40 s, 72°C for 5 min, 4°C

Table S3. Antibody information

Markers	Primary antibody	Secondary antibody
BRACHYURY	Anti-BRACHYURY,<br>goat polyclonal IgG,<br>AF2085, Santa Cruz[a],<br>Dilution 1:20 (glycerol stock)	Donkey anti-goat IgG<br>Alexa Fluor 546, A21222,<br>Life Technologies[b],<br>Dilution 1:500
CDX2	Anti-CDX2, rabbit monoclonal IgG,<br>Sab76541, Abcam[c],<br>Dilution 1:250 (glycerol stock)	Goat anti-rabbit IgG (H+l)<br>Alexa Fluor 488, A11034,<br>Life Technologies[b],<br>Dilution 1:500
cTnT	Anti-Cardiac Troponin T, Mouse<br>Monoclonal IgG1,<br>MA5-12960, Thermo Fisher[d]<br>Dilution 1:150	Goat anti-mouse IgGl(γ1),<br>Alexa Fluor 488, A21121,<br>Thermo Fisher[d]<br>Dilution 1:500

[a] Santa Cruz Biology, Inc., Santa Cruz, CA, USA
[b] Life Technologies, Carlsbad, CA, USA
[c] Abcam, Milton Road, Cambridge, UK
[d] Thermo Fisher Scientific K.K., Tokyo, Japan

References

1. Le, M.N.T., et al., *Cardiac differentiation at an initial low density of human-induced pluripotent stem cells.* In Vitro Cell Dev Biol Anim, 2018. **54**(7): p. 513-522.
2. Nishimura, K., et al., *Development of defective and persistent Sendai virus vector: a unique gene delivery/expression system ideal for cell reprogramming.* J Biol Chem, 2011. **286**(6): p. 4760-71.
3. Takahashi, K., et al., *Induction of pluripotent stem cells from adult human fibroblasts by defined factors.* cell, 2007. **131**(5): p. 861-872.
4. Miyazaki, T., et al., *Efficient Adhesion Culture of Human Pluripotent Stem Cells Using Laminin Fragments in an Uncoated Manner.* Sci Rep, 2017. **7**: p. 41165.
